# Supplementary material for: Co-evolution of tumor and immune cells during progression of multiple myeloma
Source: Nat Commun. 2021 May 7;12:2559. doi: 10.1038/s41467-021-22804-x (PMC8105337; doi:10.1038/s41467-021-22804-x)
Supplement: Supplementary file 1 — Supplementary Information [file 41467_2021_22804_MOESM1_ESM.pdf]

Co-evolution of tumor and immune cells during progression of multiple myeloma
Liu et al.

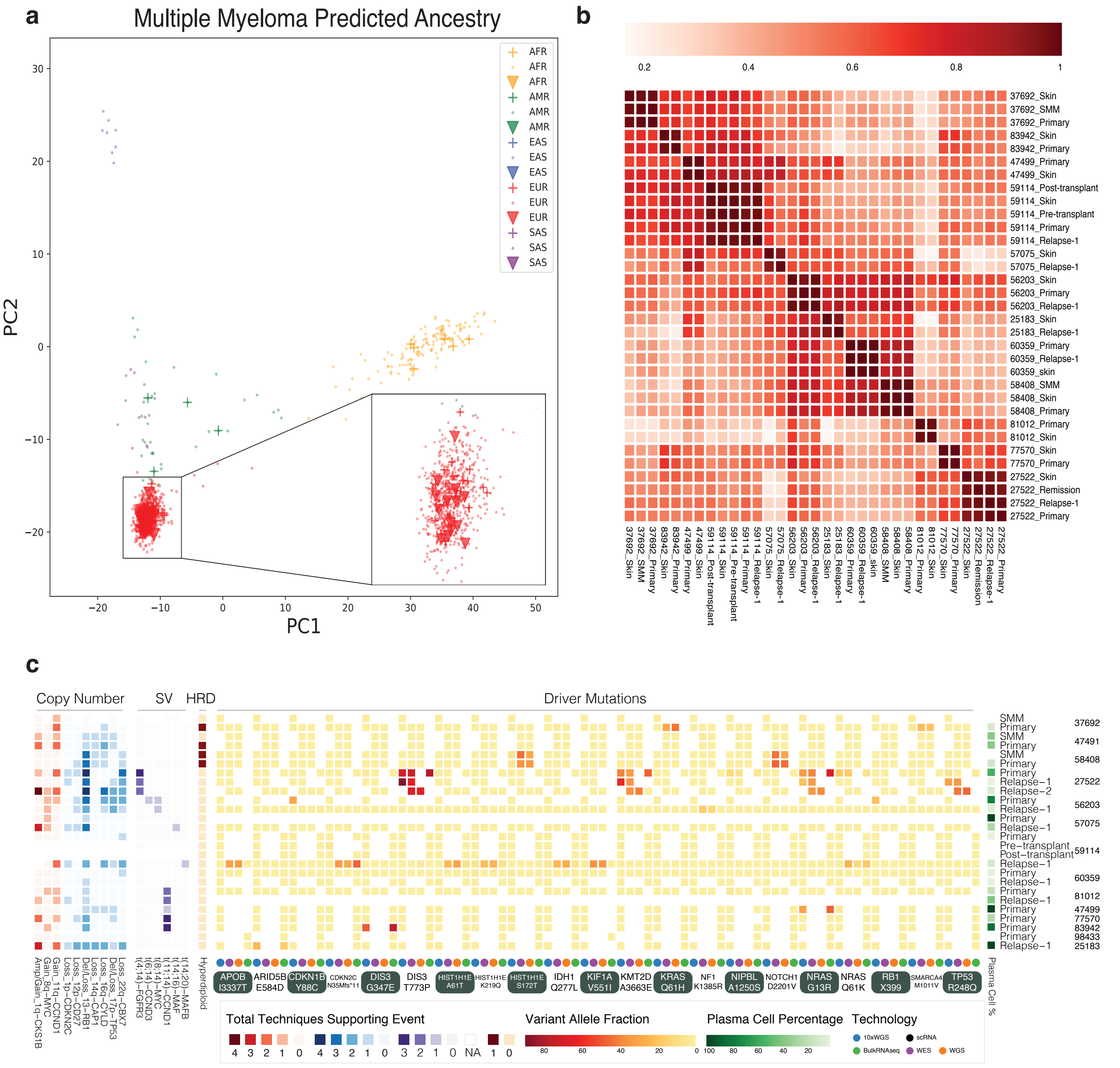

a
Multiple Myeloma Predicted Ancestry
AFR
AFR
AFR
AMR
AMR
AMR
EAS
EAS
EAS
EUR
EUR
EUR
SAS
SAS
SAS
PC2
PC1
30
20
10
0
−10
−20
−20
−10
0
10
20
30
40
50
b
0.2
0.4
0.6
0.8
1
37692_Skin
37692_SMM
37692_Primary
83942_Skin
83942_Primary
47499_Primary
47499_Skin
59114_Post-transplant
59114_Skin
59114_Pre-transplant
59114_Primary
59114_Relapse-1
57075_Skin
57075_Relapse-1
56203_Skin
56203_Primary
56203_Relapse-1
25183_Skin
25183_Relapse-1
60359_Primary
60359_Relapse-1
60359_skin
58408_SMM
58408_Skin
58408_Primary
81012_Primary
81012_Skin
77570_Skin
77570_Primary
27522_Skin
27522_Remission
27522_Relapse-1
27522_Primary
c
Copy Number
SV
HRD
Driver Mutations
Amp/Gain_1q−CKS1B
Gain_8q−MYC
Gain_11q−CCND1
Loss_1p−CDKN2C
Loss_1p−CD27
Loss_12p−CD27
Del/Loss_13−RB1
Loss_14q−CYLD
Loss_16q−CYLD
Del/Loss_17p−TP53
Loss_22q−CBX7
t(4;14)−FGFR3
t(6;14)−CCND3
t(8;14)−MYC
t(11;14)−CCND1
t(14;16)−MAF
t(14;20)−MAFB
Hyperdiploid
APOB I3337T
ARID5B E584D
CDKN1B Y88C
CDKN2C N35Mfs*11
DIS3 G347E
DIS3 T773P
HIST1H1E A61T
HIST1H1E K219Q
HIST1H1E S172T
IDH1 Q277L
KIF1A V551I
KMT2D A3663E
KRAS Q61H
NF1 K1385R
NIPBL A1250S
NOTCH1 D2201V
NRAS G13R
NRAS Q61K
RB1 X399
SMARCA4 M1011V
TP53 R248Q
Plasma Cell %
SMM
Primary
SMM
Primary
SMM
Primary
Primary
Relapse−1
Relapse−2
Primary
Relapse−1
Primary
Relapse−1
Primary
Pre−transplant
Post−transplant
Relapse−1
Primary
Relapse−1
Primary
Relapse−1
Primary
Primary
Primary
Primary
Relapse−1
37692
47491
58408
27522
56203
57075
59114
60359
81012
47499
77570
83942
98433
25183
Total Techniques Supporting Event
4 3 2 1 0 4 3 2 1 0 3 2 1 0 NA 1 0
Variant Allele Fraction
80 60 40 20 0
Plasma Cell Percentage
100 80 60 40 20
Technology
10xWGS
scRNA
BulkRNAseq
WES
WGS

Supplementary Figure 1. (a) Ancestry analysis of multiple myeloma cases. Predicted ancestry for 14 cases, along with 856 Multiple Myeloma Research Foundation cases (including 31 multiple time point cases). Analysis indicated each patient in our cohort (triangle shape) is of European ancestry, consistent with clinical records. (b) Variant allele fraction (VAF) Pearson correlations of 24 germline variants showed samples came from same patients. (c) The landscape of copy number variation (CNV), structural variation (SV), and driver mutations across from 14 patients. Copy number amplification/gain, copy number deletion/loss and SV are shown in red, blue and purple respectively with darkness of color indicating the number of techniques supporting the event. Techniques for copy number events: FISH, 10xWGS, regular WGS, WES, scRNA-seq; for SV: FISH, 10xWGS, Bulk RNA-seq, scRNA-seq. Variant allele fraction for driver mutations are shown in yellow corresponding to different sequencing techniques. Plasma cells percentage indicates the percentage of plasma cells in scRNA-seq data.

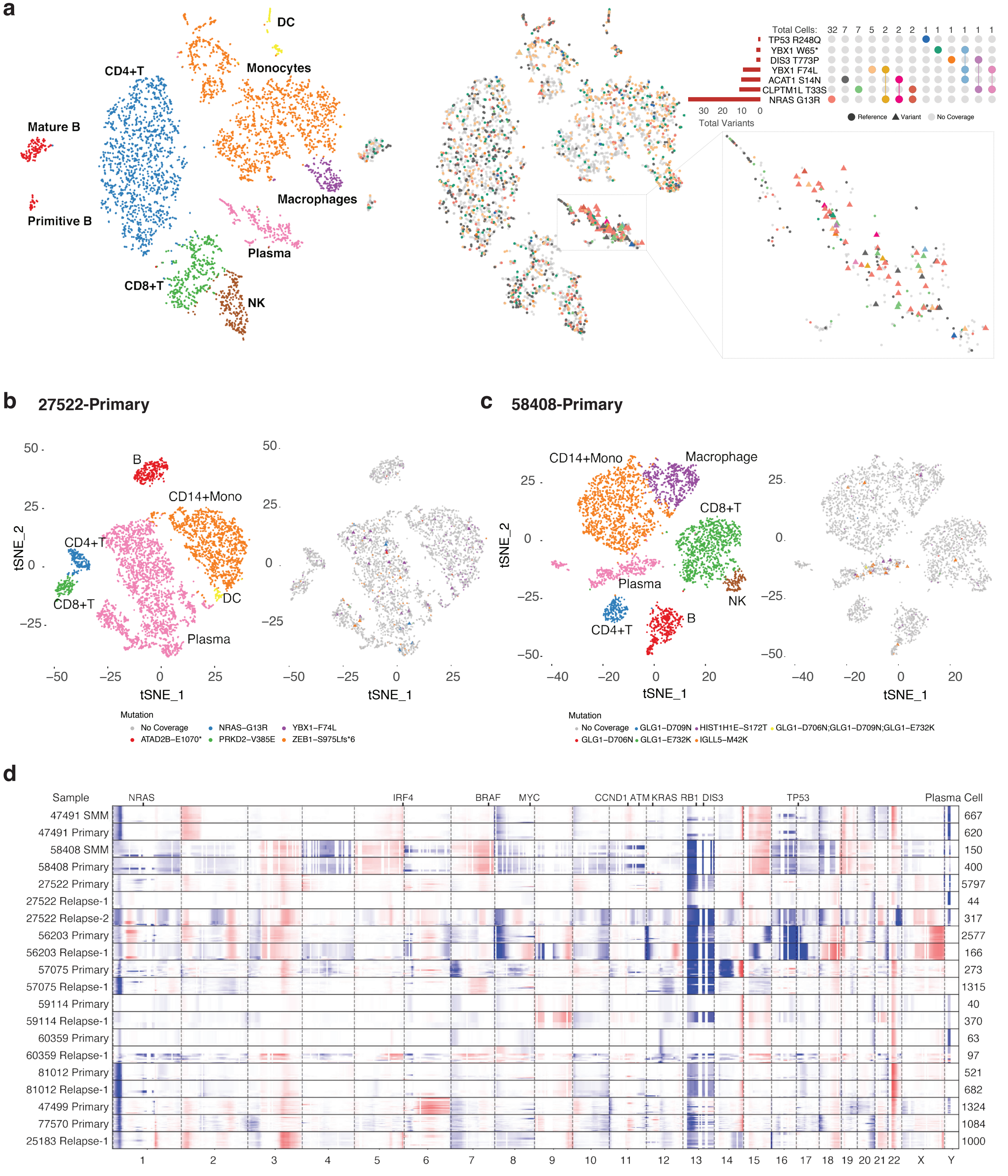
a
DC
Monocytes
CD4+T
Mature B
Primitive B
Macrophages
Plasma
CD8+T
NK
Total Cells: 32 7 7 5 2 2 2 1 1 1 1 1 1
TP53 R248Q
YBX1 W65*
DIS3 T773P
YBX1 F74L
ACAT1 S14N
CLPTM1L T33S
NRAS G13R
30 20 10 0
Total Variants
Reference
Variant
No Coverage
b
27522-Primary
B
CD14+Mono
CD4+T
CD8+T
DC
Plasma
tSNE_2
tSNE_1
Mutation
No Coverage
ATAD2B–E1070*
NRAS–G13R
PRKD2–V385E
YBX1–F74L
ZEB1–S975Lfs*6
c
58408-Primary
CD14+Mono
Macrophage
CD8+T
Plasma
NK
CD4+T
B
tSNE_2
tSNE_1
Mutation
No Coverage
GLG1–D709N
HIST1H1E–S172T
GLG1–D706N;GLG1–D709N;GLG1–E732K
GLG1–D706N
GLG1–E732K
IGLL5–M42K
d
Sample
NRAS
IRF4
BRAF
MYC
CCND1
ATM
KRAS
RB1
DIS3
TP53
Plasma Cell
47491 SMM 667
47491 Primary 620
58408 SMM 150
58408 Primary 400
27522 Primary 5797
27522 Relapse-1 44
27522 Relapse-2 317
56203 Primary 2577
56203 Relapse-1 166
57075 Primary 273
57075 Relapse-1 1315
59114 Primary 40
59114 Relapse-1 370
60359 Primary 63
60359 Relapse-1 97
81012 Primary 521
81012 Relapse-1 682
47499 Primary 1324
77570 Primary 1084
25183 Relapse-1 1000
1 2 3 4 5 6 7 8 9 10 11 12 13 14 15 16 17 18 19 20 21 22 X Y

Supplementary Figure 2. (a) Mapping reference and variant alleles to individual cell for one relapse tumor (27522 Relapse-2) and two primary tumors (b) 27522 Primary and (c) 58408 Primary. (d) Single cell CNV landscape from 20 samples.

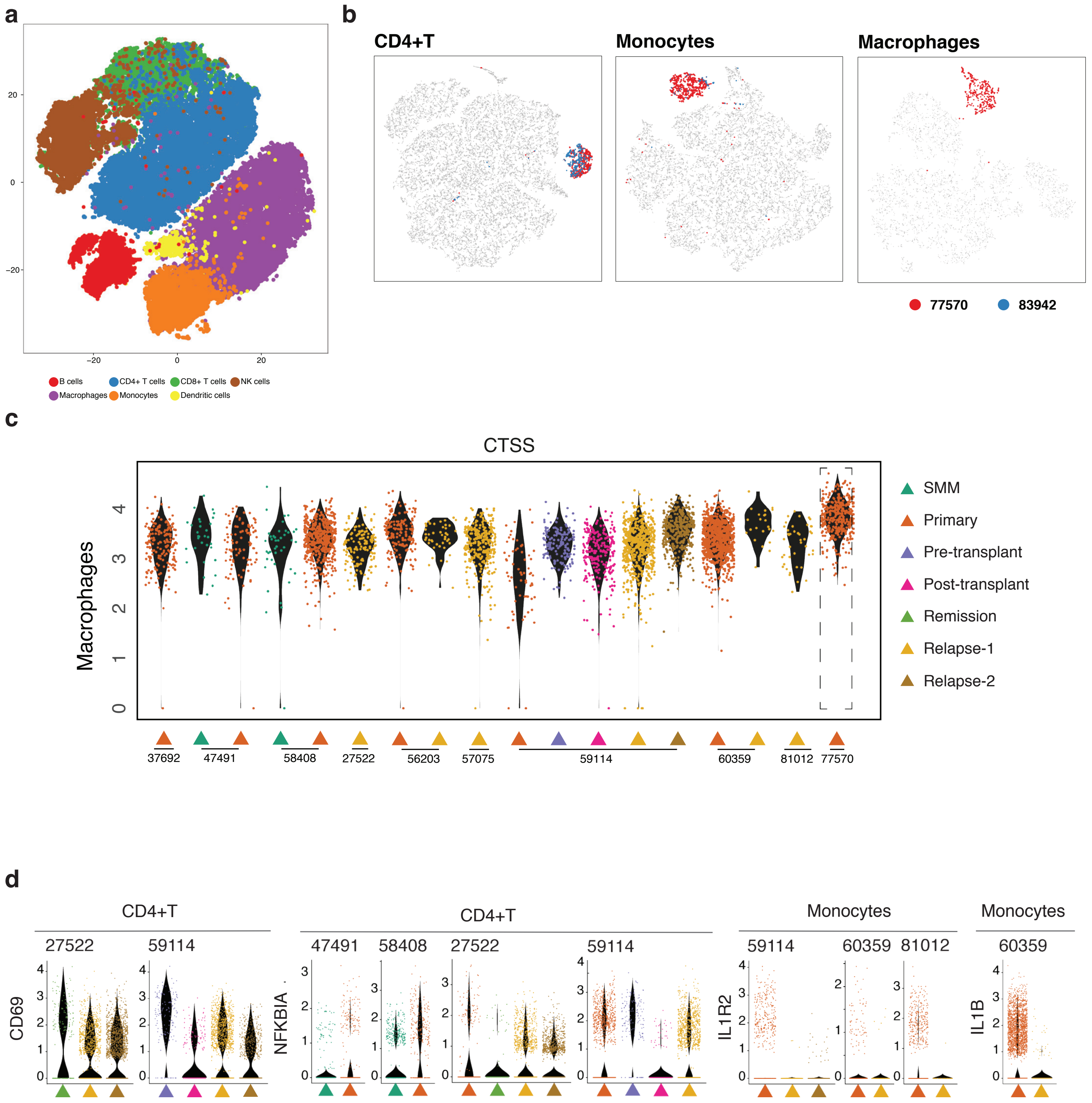

a
B cells
CD4+ T cells
CD8+ T cells
NK cells
Macrophages
Monocytes
Dendritic cells
b
CD4+T
Monocytes
Macrophages
77570
83942
c
CTSS
Macrophages
SMM
Primary
Pre-transplant
Post-transplant
Remission
Relapse-1
Relapse-2
37692
47491
58408
27522
56203
57075
59114
60359
81012
77570
d
CD4+T
27522
59114
CD69
CD4+T
47491
58408
27522
59114
NFKBIA
Monocytes
59114
60359
81012
IL1R2
Monocytes
60359
IL1B

Supplementary Figure 3. (a) t-SNE plot for non-malignant cells shows cell type dominates clustering. (b) t-SNE plot for macrophages, monocytes and CD4+ T cells, respectively. Colors indicate cells from particular samples. (c) Expression for *CTSS*, in monocytes and macrophages. (d) Expression changes over disease progression for *CD69*, *NFKBIA* in CD4+ T cells and *IL1R2*, *IL1B* in monocytes, respectively.

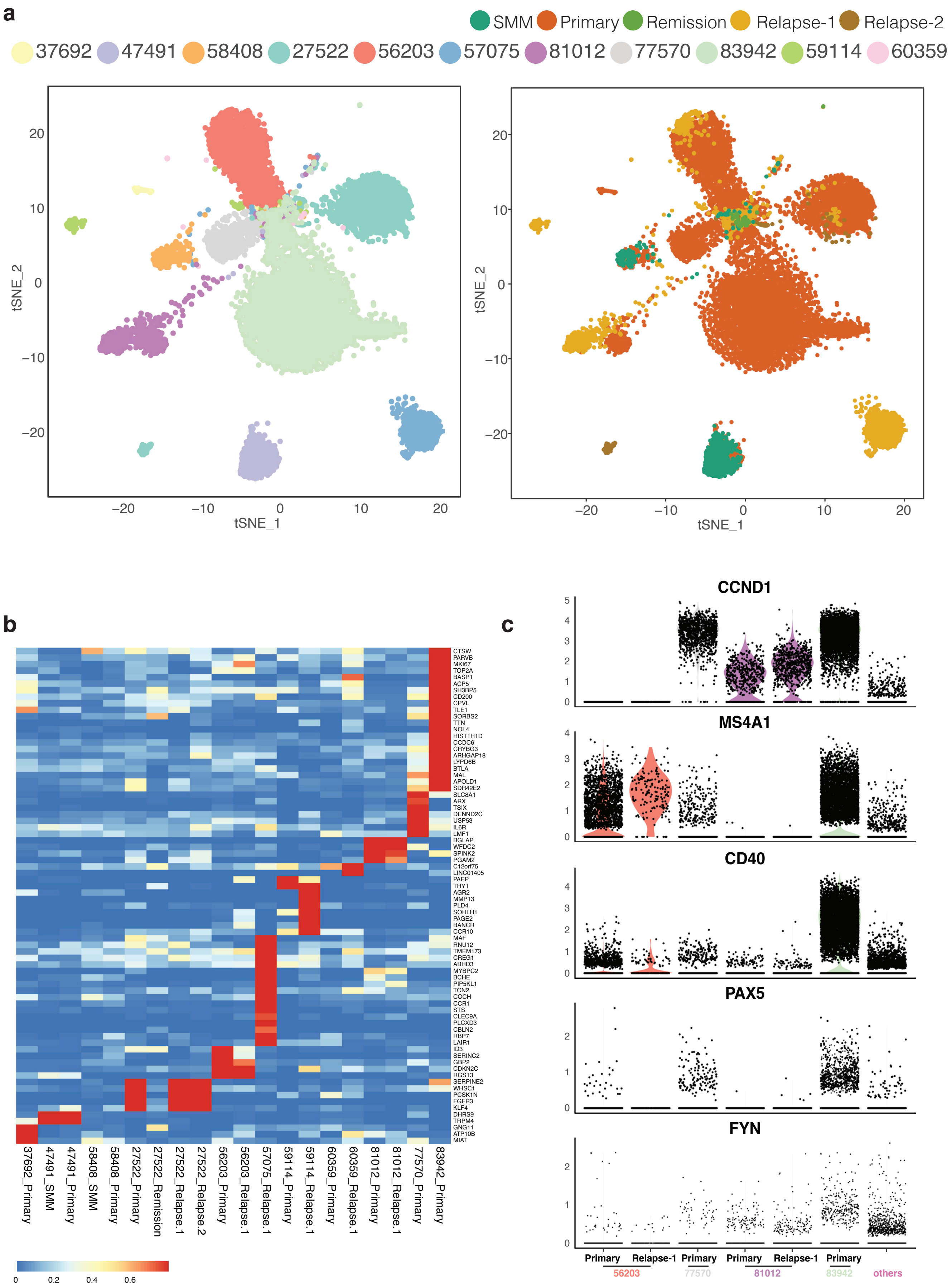
a
SMM
Primary
Remission
Relapse-1
Relapse-2
37692
47491
58408
27522
56203
57075
81012
77570
83942
59114
60359
tSNE_1
tSNE_2
b
CTSW
PARVB
MKI67
TOP2A
BASP1
ACP5
SH3BP5
CD200
CPVL
TLE1
SORBS2
TTN
NOL4
HIST1H1D
CCDC6
CRYBG3
ARHGAP18
LYPD6B
BTLA
MAL
APOLD1
SDR42E2
SLC8A1
ARX
TSIX
DENND2C
USP53
IL6R
LMF1
BGLAP
WFDC2
SPINK2
PGAM2
C12orf75
LINC01405
PAEP
THY1
AGR2
MMP13
PLD4
SOHLH1
PAGE2
BANCR
CCR10
MAF
RNU12
TMEM173
CREG1
ABHD3
MYBPC2
BCHE
PIP5KL1
TCN2
COCH
CCR1
STS
CLEC9A
PLCXD3
CBLN2
RBP7
LAIR1
ID3
SERINC2
GBP2
CDKN2C
RGS13
SERPINE2
WHSC1
PCSK1N
FGFR3
KLF4
DHRS9
TRPM4
GNG11
ATP10B
MIAT
37692_Primary
47491_SMM
47491_Primary
58408_SMM
58408_Primary
27522_Primary
27522_Remission
27522_Relapse.1
27522_Relapse.2
56203_Primary
56203_Relapse.1
57075_Relapse.1
59114_Primary
59114_Relapse.1
60359_Primary
60359_Relapse.1
81012_Primary
81012_Relapse.1
77570_Primary
83942_Primary
c
CCND1
MS4A1
CD40
PAX5
FYN
Primary
Relapse-1
Primary
Primary
Relapse-1
Primary
56203
77570
81012
83942
others

Supplementary Figure 4. (a) t-SNE plots showing malignant cells from eleven patients reveal tumor-specific clusters, colored by patient (left) and stage (right). (b) Heatmap showing genes with individual-specific expression pattern (c) Violin plot showing the expression pattern of plasma cells for samples potentially within CD1/CD2 groups.

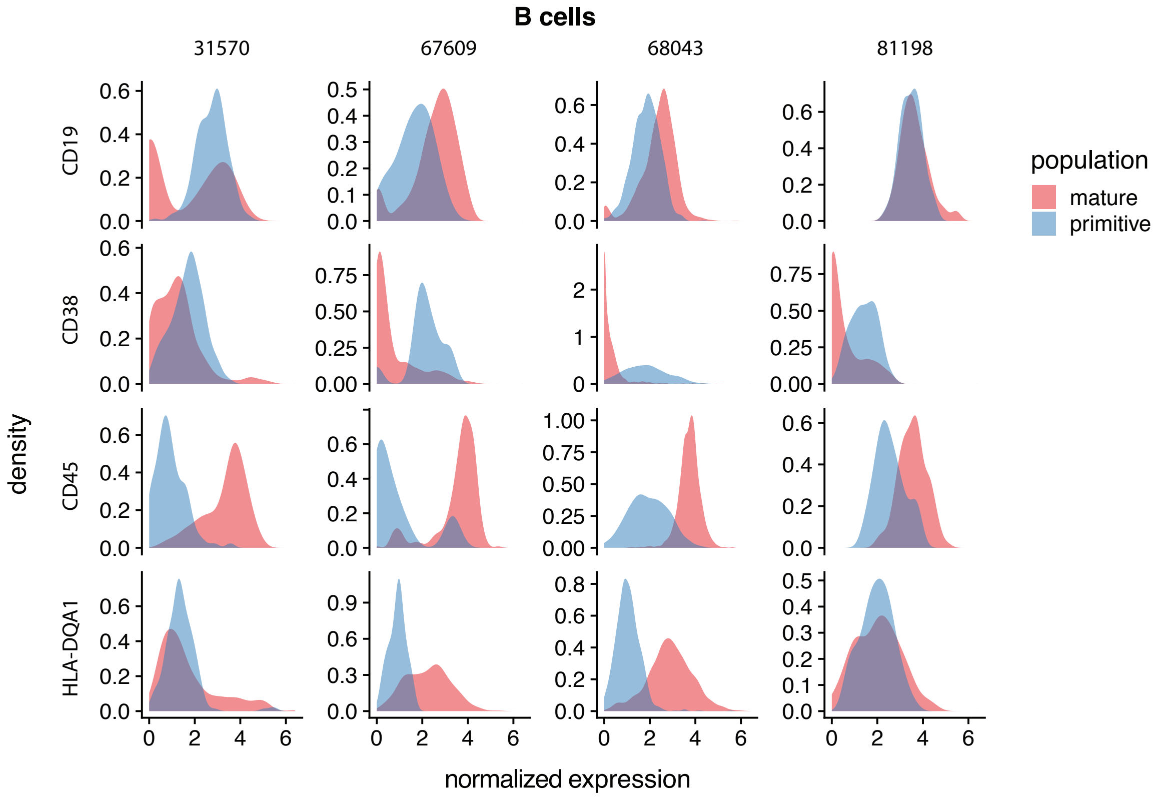

B cells
31570
67609
68043
81198
CD19
CD38
CD45
HLA-DQA1
density
normalized expression
population
mature
primitive

Supplementary Figure 5. CyTOF profiling shows HLA-DQA1 is differentially expressed between primitive and mature B cells.

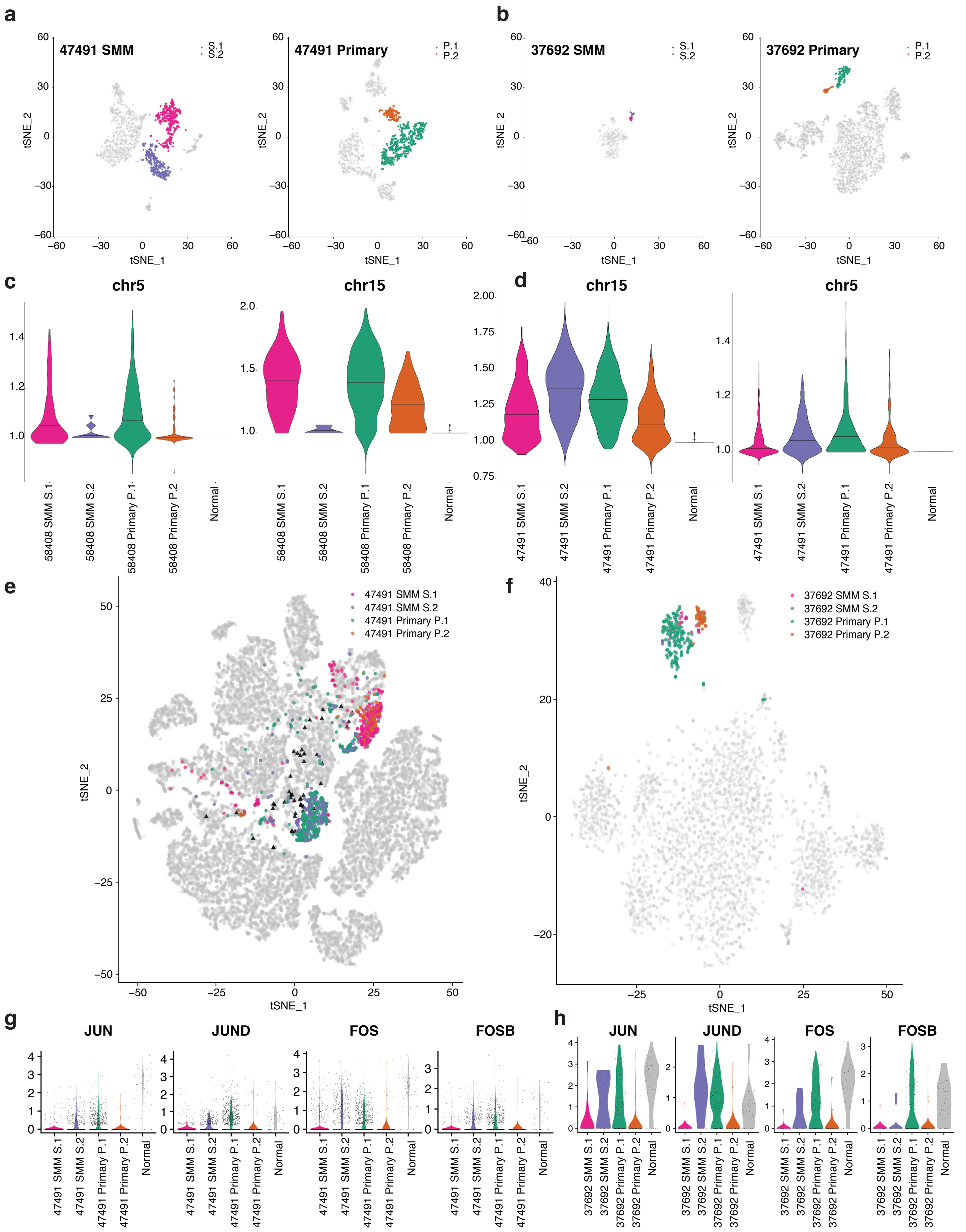
a
47491 SMM
S.1
S.2
47491 Primary
P.1
P.2
tSNE_1
tSNE_2
b
37692 SMM
S.1
S.2
37692 Primary
P.1
P.2
c
chr5
chr15
58408 SMM S.1
58408 SMM S.2
58408 Primary P.1
58408 Primary P.2
Normal
d
chr15
chr5
47491 SMM S.1
47491 SMM S.2
47491 Primary P.1
47491 Primary P.2
Normal
e
47491 SMM S.1
47491 SMM S.2
47491 Primary P.1
47491 Primary P.2
f
37692 SMM S.1
37692 SMM S.2
37692 Primary P.1
37692 Primary P.2
g
JUN
JUND
FOS
FOSB
h
JUN
JUND
FOS
FOSB
37692 SMM S.1
37692 SMM S.2
37692 Primary P.1
37692 Primary P.2
Normal

Supplementary Figure 6. (a) Plasma subpopulations identified in Patient 47491 SMM (47491_1) and Primary (47491_2). (b) Plasma subpopulations identified in Patient 37692 SMM (37692_1) and Primary (37692_2). (c) Copy number alterations in different plasma cell populations in Patient 47491. (d) Copy number alterations in different plasma cell populations in Patient 58408. (e) Mapping of plasma cell subpopulations from Patient 47491 SMM and Primary samples onto the integrated B cell and plasma cell t-SNE plot from all samples. (f) Mapping of plasma cell subpopulations from Patient 37692 SMM and Primary samples to their integrated t-SNE. (g) *JUN* and *FOS* expression in Patient 47491. (h) *JUN* and *FOS* expression in Patient 37692.

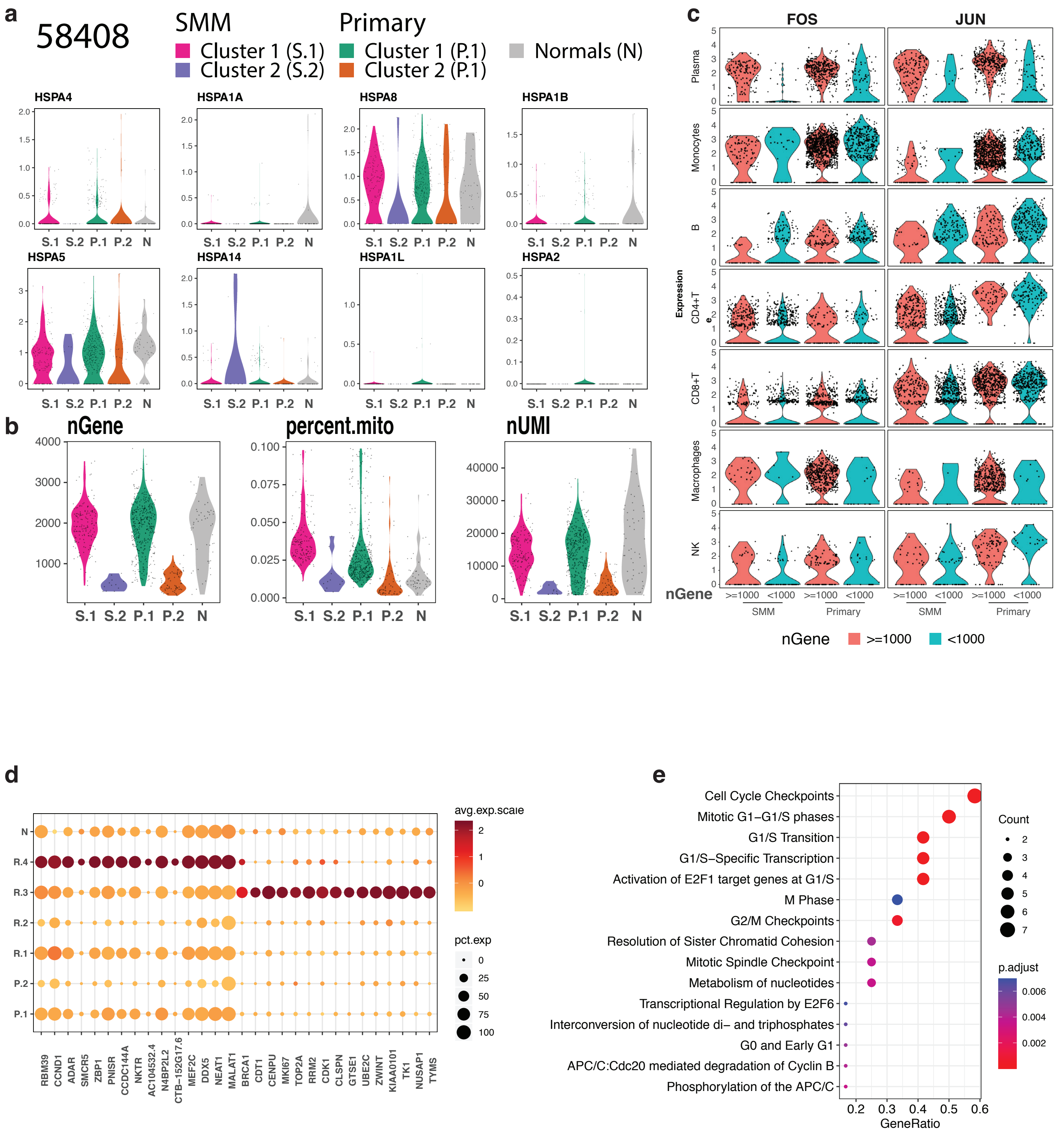
a
58408
SMM
Cluster 1 (S.1)
Cluster 2 (S.2)
Primary
Cluster 1 (P.1)
Cluster 2 (P.1)
Normals (N)
HSPA4
HSPA1A
HSPA8
HSPA1B
HSPA5
HSPA14
HSPA1L
HSPA2
S.1 S.2 P.1 P.2 N
b
nGene
percent.mito
nUMI
c
FOS
JUN
Plasma
Monocytes
B
CD4+T
CD8+T
Macrophages
NK
Expression
nGene
>=1000 <1000 >=1000 <1000
SMM
Primary
nGene >=1000 <1000
d
avg.exp.scale
pct.exp
N
R.4
R.3
R.2
R.1
P.2
P.1
RBM39 CCND1 ADAR SMCR5 ZBP1 PNISR CCDC144A NKTR AC104532.4 N4BP2L2 CTB−152G17.6 MEF2C DDX5 NEAT1 MALAT1 BRCA1 CDT1 CENPU MKI67 TOP2A RRM2 CDK1 CLSPN GTSE1 UBE2C ZWINT KIAA0101 TK1 NUSAP1 TYMS
e
Cell Cycle Checkpoints
Mitotic G1−G1/S phases
G1/S Transition
G1/S−Specific Transcription
Activation of E2F1 target genes at G1/S
M Phase
G2/M Checkpoints
Resolution of Sister Chromatid Cohesion
Mitotic Spindle Checkpoint
Metabolism of nucleotides
Transcriptional Regulation by E2F6
Interconversion of nucleotide di− and triphosphates
G0 and Early G1
APC/C:Cdc20 mediated degradation of Cyclin B
Phosphorylation of the APC/C
GeneRatio
Count
p.adjust

Supplementary Figure 7. (a) Expression of heat shock genes in different subclusters of plasma cells in Patient 58408 (SMM and Primary). (b) Quality control parameters in subclusters of plasma cells in Patient 58408. (c) Violin Plot showing expression of *FOS* and *JUN* in in different types of cells for Patient 58408. Cells are separated by nGene>=1000 (red) and nGene < 1000 (blue). (d) Dot Plot showing the expression of genes specifically upregulated in 81012 R.3 and R.4. (e) Pathway enrichment analysis for genes upregulated in case 81012 R.3. The x axis indicates the ratio of genes present in the corresponding pathway gene list. Dot size indicate the number of genes present in the corresponding pathway; color indicates the adjusted p value. Enrichment analysis is carried out by performing the hypergeometric test, p value is then adjusted by Benjamini-Hochberg (BH) correction.

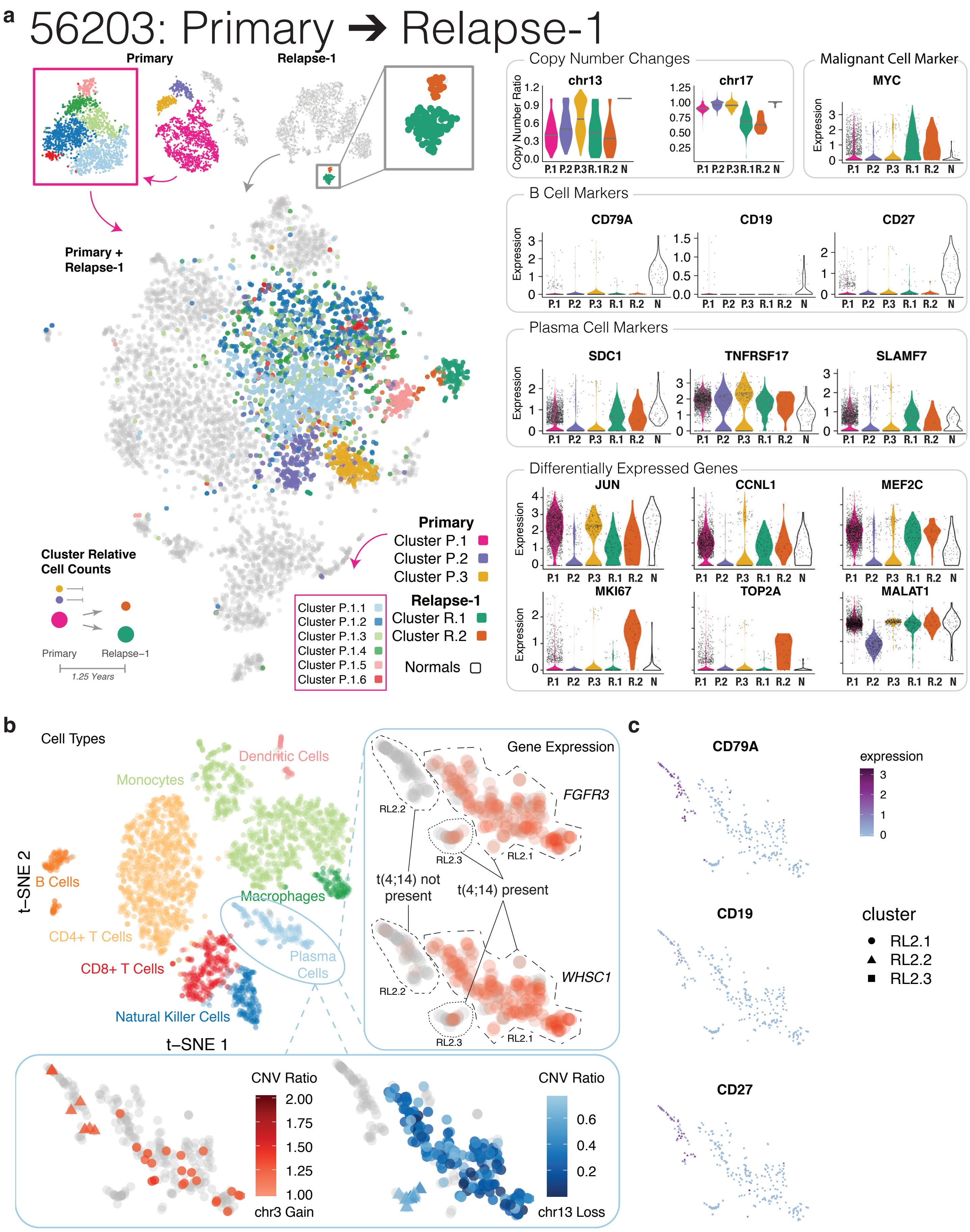
a
56203: Primary ➔ Relapse-1
Primary
Relapse-1
Primary + Relapse-1
Copy Number Changes
chr13
chr17
Copy Number Ratio
Malignant Cell Marker
MYC
Expression
B Cell Markers
CD79A
CD19
CD27
Plasma Cell Markers
SDC1
TNFRSF17
SLAMF7
Differentially Expressed Genes
JUN
CCNL1
MEF2C
MKI67
TOP2A
MALAT1
P.1 P.2 P.3 R.1 R.2 N
Cluster Relative Cell Counts
Primary
Relapse−1
1.25 Years
Primary
Cluster P.1
Cluster P.2
Cluster P.3
Relapse-1
Cluster R.1
Cluster R.2
Normals
Cluster P.1.1
Cluster P.1.2
Cluster P.1.3
Cluster P.1.4
Cluster P.1.5
Cluster P.1.6
b
Cell Types
Dendritic Cells
Monocytes
B Cells
CD4+ T Cells
CD8+ T Cells
Natural Killer Cells
Macrophages
Plasma Cells
t−SNE 1
t−SNE 2
Gene Expression
FGFR3
WHSC1
RL2.1
RL2.2
RL2.3
t(4;14) not present
t(4;14) present
CNV Ratio
2.00
1.75
1.50
1.25
1.00
chr3 Gain
0.6
0.4
0.2
chr13 Loss
c
CD79A
CD19
CD27
expression
cluster
RL2.1
RL2.2
RL2.3

Supplementary Figure 8. (a) Patient 56203 from the primary stage, with three plasma cell subpopulations (P.1, P.2, and P.3), to the relapse stage, with two plasma cell subpopulations (R.1 and R.2). (b) t-SNE plot showing the expression of *WHSC1*/ *FGFR3* and copy number profile in terms of chromosome 3 gain and chromosome 13 loss, in each subcluster of plasma cells in the relapse-2 sample of case 27522. (c) Expression of B cell markers in plasma cells from Patient 27522 Relapse-2.

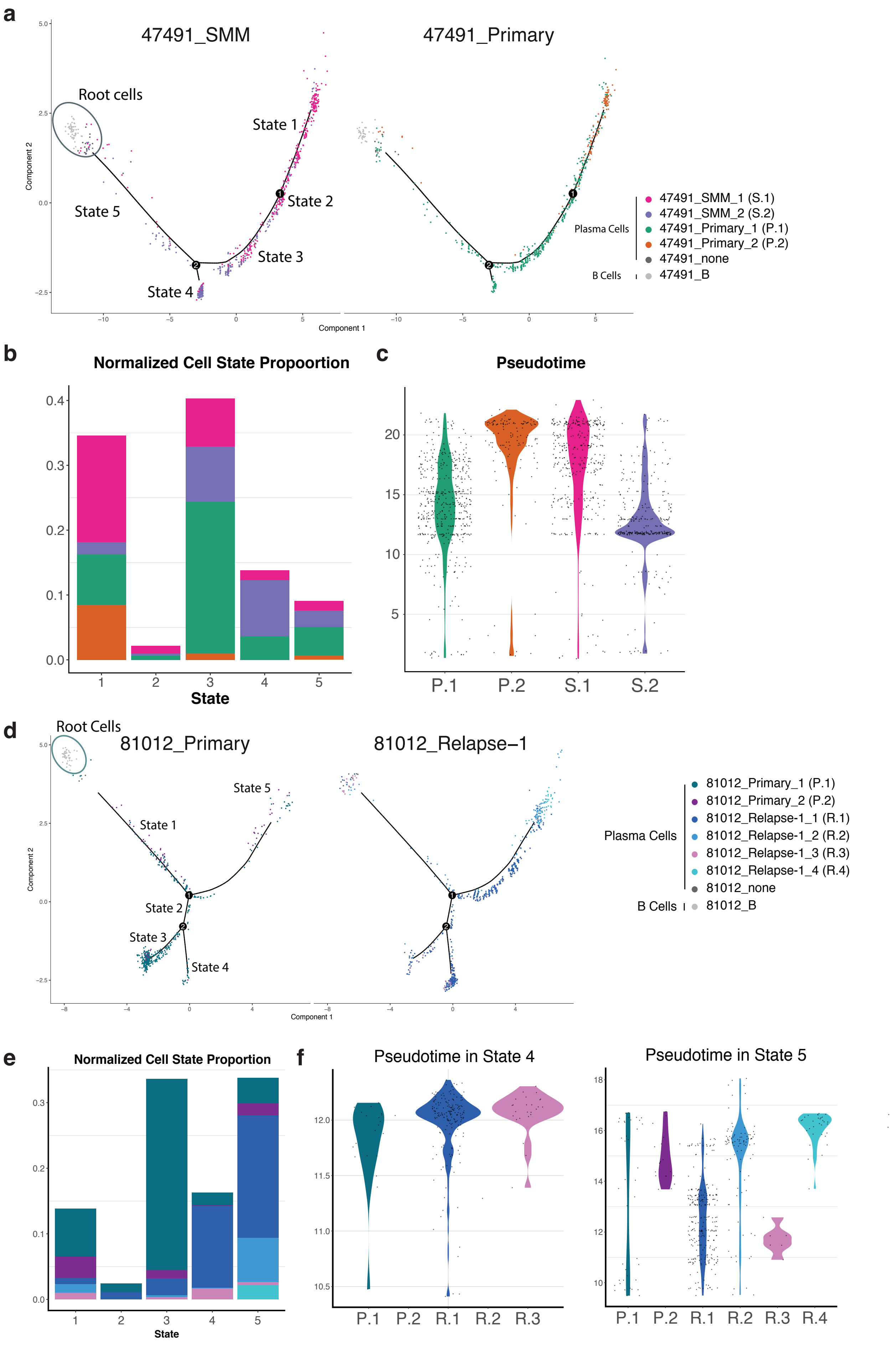

a
47491_SMM
47491_Primary
Root cells
State 1
State 2
State 3
State 4
State 5
Component 2
Component 1
Plasma Cells
B Cells
47491_SMM_1 (S.1)
47491_SMM_2 (S.2)
47491_Primary_1 (P.1)
47491_Primary_2 (P.2)
47491_none
47491_B
b
Normalized Cell State Propoortion
State
c
Pseudotime
P.1
P.2
S.1
S.2
d
Root Cells
81012_Primary
81012_Relapse−1
State 1
State 2
State 3
State 4
State 5
Component 2
Component 1
Plasma Cells
B Cells
81012_Primary_1 (P.1)
81012_Primary_2 (P.2)
81012_Relapse-1_1 (R.1)
81012_Relapse-1_2 (R.2)
81012_Relapse-1_3 (R.3)
81012_Relapse-1_4 (R.4)
81012_none
81012_B
e
Normalized Cell State Proportion
State
f
Pseudotime in State 4
Pseudotime in State 5
P.1
P.2
R.1
R.2
R.3
R.4

Supplementary Figure 9. (a) Trajectory analysis for B and Plasma cells in Patient 47491; cells are colored by cell type (B/plasma) and subcluster information (corresponding to subcluster information from extended data figure 6). Left: cells from SMM; right: cells from primary diagnosis. (b) Normalized proportion for plasma cell subclusters within each state for Patient 47491. (c) Violin plot showing pseudotime distribution across states for plasma cell subclusters in Patient 47491. (d) Similar as (a) except that the case shown is from Patient 81012 and two time points are from primary diagnosis and relapse, respectively. (e-f) Normalized plasma cell subcluster proportion and pseudotime distribution for Patient 81012, similar to (b)-(c).

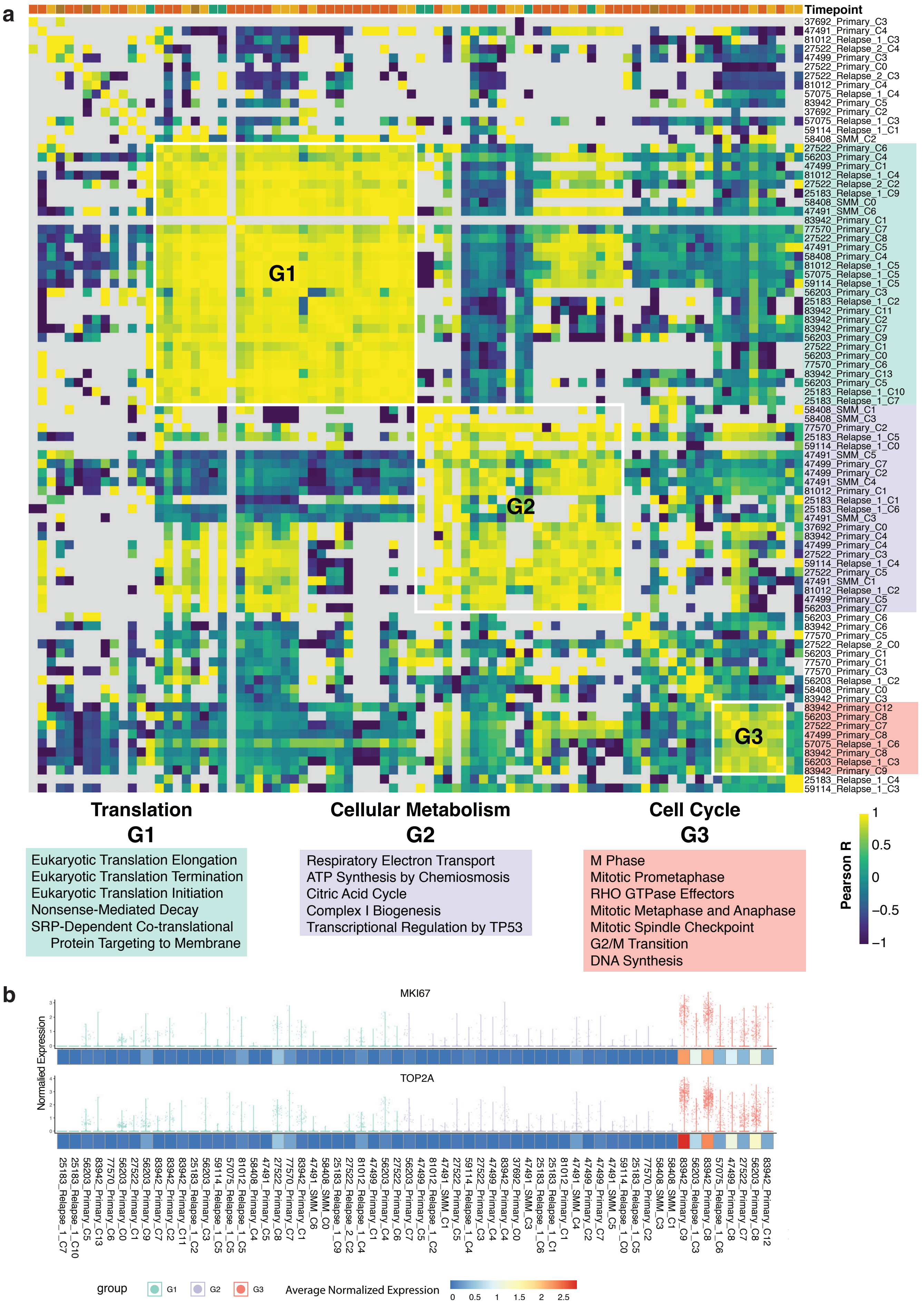
a
Timepoint
37692_Primary_C3
47491_Primary_C4
81012_Relapse_1_C3
27522_Relapse_2_C4
47499_Primary_C3
27522_Primary_C0
27522_Relapse_2_C3
81012_Primary_C4
57075_Relapse_1_C4
83942_Primary_C5
37692_Primary_C2
57075_Relapse_1_C3
59114_Relapse_1_C1
58408_SMM_C2
27522_Primary_C6
56203_Primary_C4
47499_Primary_C1
81012_Relapse_1_C4
27522_Relapse_2_C2
25183_Relapse_1_C9
58408_SMM_C0
47491_SMM_C6
83942_Primary_C1
77570_Primary_C7
27522_Primary_C8
47491_Primary_C5
58408_Primary_C4
81012_Relapse_1_C5
57075_Relapse_1_C5
59114_Relapse_1_C5
56203_Primary_C3
25183_Relapse_1_C2
83942_Primary_C11
83942_Primary_C2
83942_Primary_C7
56203_Primary_C9
27522_Primary_C1
56203_Primary_C0
77570_Primary_C6
83942_Primary_C13
56203_Primary_C5
25183_Relapse_1_C10
25183_Relapse_1_C7
58408_SMM_C1
58408_SMM_C3
77570_Primary_C2
25183_Relapse_1_C5
59114_Relapse_1_C0
47491_SMM_C5
47499_Primary_C7
47499_Primary_C2
47491_SMM_C4
81012_Primary_C1
25183_Relapse_1_C1
25183_Relapse_1_C6
47491_SMM_C3
37692_Primary_C0
83942_Primary_C4
47499_Primary_C4
27522_Primary_C3
59114_Relapse_1_C4
27522_Primary_C5
47491_SMM_C1
81012_Relapse_1_C2
47499_Primary_C5
56203_Primary_C7
56203_Primary_C6
83942_Primary_C6
77570_Primary_C5
27522_Relapse_2_C0
56203_Primary_C1
77570_Primary_C1
77570_Primary_C3
56203_Relapse_1_C2
58408_Primary_C0
83942_Primary_C3
83942_Primary_C12
56203_Primary_C8
27522_Primary_C7
47499_Primary_C8
57075_Relapse_1_C6
83942_Primary_C8
56203_Relapse_1_C3
83942_Primary_C9
25183_Relapse_1_C4
59114_Relapse_1_C3
G1
G2
G3
Translation
G1
Eukaryotic Translation Elongation
Eukaryotic Translation Termination
Eukaryotic Translation Initiation
Nonsense-Mediated Decay
SRP-Dependent Co-translational
Protein Targeting to Membrane
Cellular Metabolism
G2
Respiratory Electron Transport
ATP Synthesis by Chemiosmosis
Citric Acid Cycle
Complex I Biogenesis
Transcriptional Regulation by TP53
Cell Cycle
G3
M Phase
Mitotic Prometaphase
RHO GTPase Effectors
Mitotic Metaphase and Anaphase
Mitotic Spindle Checkpoint
G2/M Transition
DNA Synthesis
Pearson R
1
0.5
0
−0.5
−1
b
MKI67
TOP2A
Normalied Expression
25183_Relapse_1_C7
25183_Relapse_1_C10
56203_Primary_C5
83942_Primary_C13
77570_Primary_C6
56203_Primary_C0
27522_Primary_C1
56203_Primary_C9
83942_Primary_C7
83942_Primary_C2
83942_Primary_C11
25183_Relapse_1_C2
56203_Primary_C3
59114_Relapse_1_C5
57075_Relapse_1_C5
81012_Relapse_1_C5
58408_Primary_C4
47491_Primary_C5
27522_Primary_C8
77570_Primary_C7
83942_Primary_C1
47491_SMM_C6
58408_SMM_C0
25183_Relapse_1_C9
27522_Relapse_2_C2
81012_Relapse_1_C4
47499_Primary_C1
56203_Primary_C4
27522_Primary_C6
56203_Primary_C7
47499_Primary_C5
81012_Relapse_1_C2
47491_SMM_C1
27522_Primary_C5
59114_Relapse_1_C4
27522_Primary_C3
47499_Primary_C4
83942_Primary_C4
37692_Primary_C0
47491_SMM_C3
25183_Relapse_1_C6
25183_Relapse_1_C1
81012_Primary_C1
47491_SMM_C4
47499_Primary_C2
47499_Primary_C7
47491_SMM_C5
59114_Relapse_1_C0
25183_Relapse_1_C5
77570_Primary_C2
58408_SMM_C3
58408_SMM_C1
83942_Primary_C9
56203_Relapse_1_C3
83942_Primary_C8
57075_Relapse_1_C6
47499_Primary_C8
27522_Primary_C7
56203_Primary_C8
83942_Primary_C12
group
G1
G2
G3
Average Normalized Expression
0
0.5
1
1.5
2
2.5

Supplementary Figure 10. (a) Unsupervised clustering of sub-populations of plasma cells from 16 MM patients (x-axis and y-axis) based on correlation with significant pathways. Three major groups emerged (G1, G2, G3) that contained significantly correlated sub-populations based on shared pathways. The highly significant pathways from three groups displayed in the figure (Translation, Cellular Metabolism, Cell Cycle). The highly significant pathways from three groups displayed in the figure (Translation, Cellular Metabolism, Cell Cycle) contained significantly correlated sub-populations based on shared pathways. (b) Expression of two proliferation markers *MKI67* and *TOP2A* in different subclusters of plasma cells belong to three major groups. Each dot shows the normalized expression for individual cells; heatmap shows the average expression for each subclsuter.

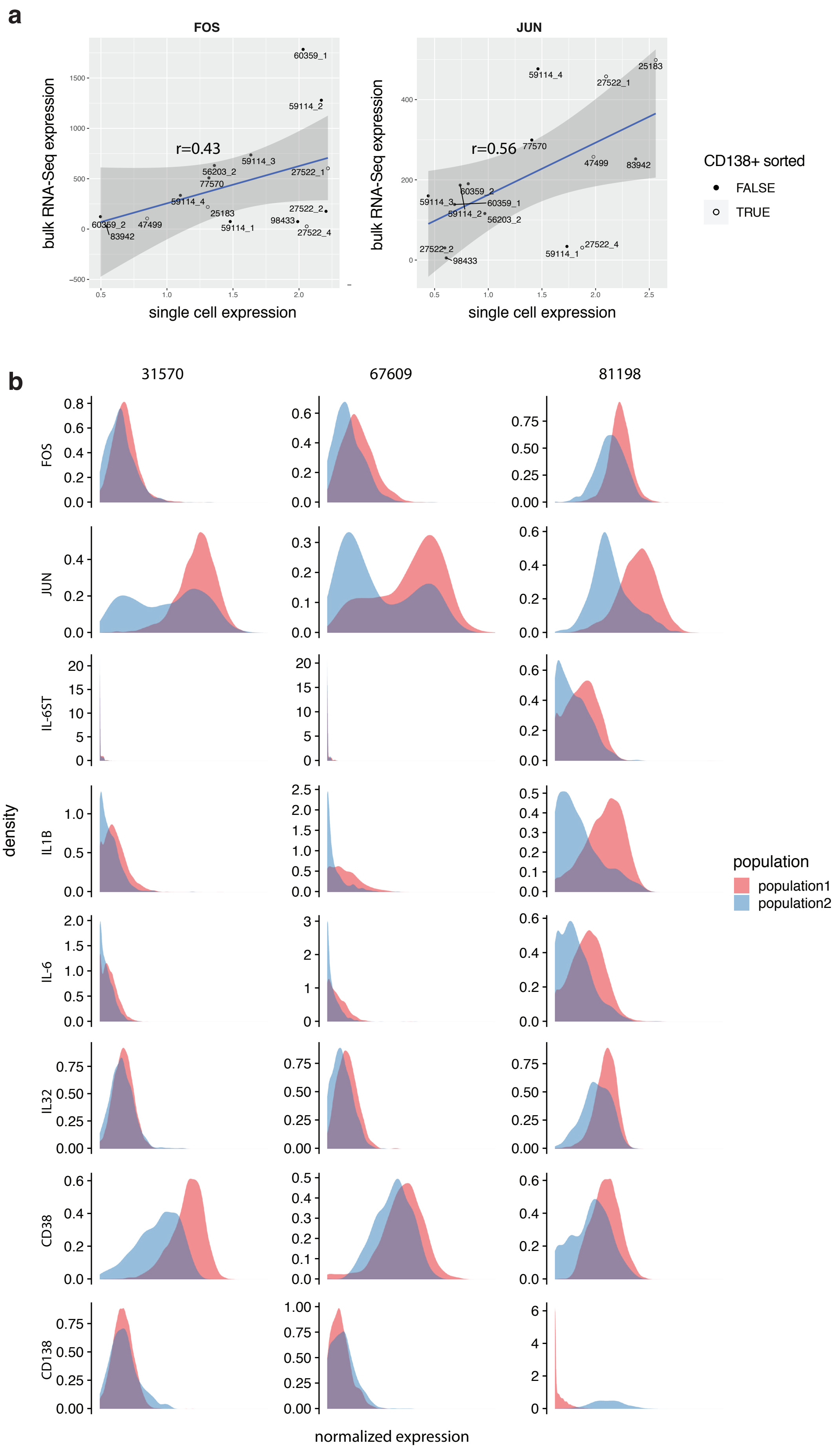
a
FOS
JUN
bulk RNA-Seq expression
single cell expression
r=0.43
r=0.56
60359_1
59114_2
59114_3
56203_2
77570
27522_1
59114_4
25183
27522_2
98433
47499
60359_2
83942
59114_1
27522_4
25183
59114_4
27522_1
77570
47499
83942
60359_2
59114_3
60359_1
59114_2
56203_2
27522_2
98433
59114_1
27522_4
CD138+ sorted
FALSE
TRUE
b
31570
67609
81198
FOS
JUN
IL-6ST
IL1B
IL-6
IL32
CD38
CD138
density
population
population1
population2
normalized expression

Supplementary Figure 11. (a) Correlation of single cell average expression and bulk RNA-Seq expression for each sample for FOS and JUN. Solid dot indicates unsorted samples. Blank dot indicates samples for bulk RNA-Seq is positively sorted for CD138, and single cell average expression in this scenario is calculated with plasma cells only. (b) CyTOF profiling of genes of interest in two manually-gated populations of plasma cells for three samples with good viability.
