# Supplementary material for: Co-evolution of tumor and immune cells during progression of multiple myeloma
Source: Nat Commun. 2021 May 7;12:2559. doi: 10.1038/s41467-021-22804-x (PMC8105337; doi:10.1038/s41467-021-22804-x)
Supplement: Supplementary file 3 — Description of Additional Supplementary Files [file 41467_2021_22804_MOESM3_ESM.pdf]

Description of Additional Supplementary Files

File Name: Supplementary Data 1
Description: Patient clinical information, data quality control and preliminary analysis data

File Name: Supplementary Data 2
Description: Summary of single cell mutation mapping

File Name: Supplementary Data 3
Description: Gene expression summary for single cell data

File Name: Supplementary Data 4
Description: Summary of differentially expressed genes

File Name: Supplementary Data 5
Description: Somatic variant allele frequencies across stages

File Name: Supplementary Data 6
Description: CyTOF panel summary
