# Supplementary material for: Co-evolution of tumor and immune cells during progression of multiple myeloma
Source: Nat Commun. 2021 May 7;12:2559. doi: 10.1038/s41467-021-22804-x (PMC8105337; doi:10.1038/s41467-021-22804-x)
Supplement: Supplementary file 10 — Reporting Summary [file 41467_2021_22804_MOESM10_ESM.pdf]

nature research

Corresponding author(s): Li Ding

Last updated by author(s): Jan 31, 2021

Reporting Summary

Nature Research wishes to improve the reproducibility of the work that we publish. This form provides structure for consistency and transparency in reporting. For further information on Nature Research policies, see our Editorial Policies and the Editorial Policy Checklist.

Statistics

For all statistical analyses, confirm that the following items are present in the figure legend, table legend, main text, or Methods section.

n/a	Confirmed	
[ ]	[x]	The exact sample size (*n*) for each experimental group/condition, given as a discrete number and unit of measurement
[ ]	[x]	A statement on whether measurements were taken from distinct samples or whether the same sample was measured repeatedly
[ ]	[x]	The statistical test(s) used AND whether they are one- or two-sided<br>*Only common tests should be described solely by name; describe more complex techniques in the Methods section.*
[x]	[ ]	A description of all covariates tested
[ ]	[x]	A description of any assumptions or corrections, such as tests of normality and adjustment for multiple comparisons
[ ]	[x]	A full description of the statistical parameters including central tendency (e.g. means) or other basic estimates (e.g. regression coefficient) AND variation (e.g. standard deviation) or associated estimates of uncertainty (e.g. confidence intervals)
[ ]	[x]	For null hypothesis testing, the test statistic (e.g. *F*, *t*, *r*) with confidence intervals, effect sizes, degrees of freedom and *P* value noted<br>*Give P values as exact values whenever suitable.*
[x]	[ ]	For Bayesian analysis, information on the choice of priors and Markov chain Monte Carlo settings
[ ]	[x]	For hierarchical and complex designs, identification of the appropriate level for tests and full reporting of outcomes
[ ]	[x]	Estimates of effect sizes (e.g. Cohen's *d*, Pearson's *r*), indicating how they were calculated

Our web collection on statistics for biologists contains articles on many of the points above.

Software and code

Policy information about availability of computer code

Data collection

Somatic mutation detection
Somatic variants were called by our SomaticWrapper pipeline, which includes four established bioinformatic tools, namely Strelka, Mutect, VarScan2 (2.3.83), and Pindel (0.2.54). We retained SNVs and INDELs using the following strategy: keep SNVs called by any 2 callers among Mutect, VarScan, and Strelka and INDELs called by any 2 callers among VarScan, Strelka, and Pindel. For these merged SNVs and INDELs, we applied coverage cut-offs of 14X and 8X for tumor and normal, respectively. We also filtered SNVs and INDELs with a high-pass variant allele fraction (VAF) of 0.05 in tumor and a low-pass VAF of 0.02 in normal. The SomaticWrapper pipeline is freely available from GitHub at https://github.com/ding-lab/somaticwrapper.

Copy Number and Structural Variation Detection
We used BIC-seq2, a read-depth-based CNV calling algorithm to detect somatic copy number variations (CNVs) using standard WGS tumor samples and paired skin 10xWGS data (human genome GRCh38 reference). The procedure involves 1) retrieving all uniquely mapped reads from the tumor and paired skin BAM files, 2) removing biases by normalization (NBICseq-norm_v0.2.4), 3) detecting CNV based on normalized data (NBICseq-seg_v0.7.2) with BIC-seq2 parameters set as --lambda=90 --detail --noscale --control. In WES data, we used CNVkit (v0.9.4) to compare our tumor samples to a background panel of normals. For scRNA-seq data, we used inferCNV (v0.8.2).
Somatic structural variants (SVs) were detected by Manta using tumor/normal sample pairs of standard WGS and paired skin 10xWGS. To filter false positive SVs, we removed events with somatic score < 30 and junction somatic score < 30.

Analysis of 10x Genomics whole genome sequencing data
The proprietary Long Ranger system (v2.2.2) from 10x Genomics was used for preliminary analysis, including demultiplexing cDNA libraries into FASTQ files and aligning reads to the human genome reference GRCh38 (GRCh38-2.1.0). To call variants using Long Ranger, we used --vcmode with GATK (version 3.7.0-gcfedb67). Long Ranger phasing quality metrics were extracted from the summary output file associated with each sample. For haplotype analysis of somatic variants, we relied on phase information of germline variation from surrounding loci on the same set of linked-reads.

Analysis of bulk RNA-seq data
Gene expression was estimated using Kallisto (v0.43.1) and gene fusions were detected using STAR-Fusion (v1.4.0). We used GRCh38_v27_CTAT_lib_Feb092018 from the STAR-fusion website as the human reference and corresponding GENCODE annotation sets.

Analysis of scRNA-seq data
For single cell RNA-seq analysis, the proprietary software tool Cell Ranger (v2.1.1) from 10x Genomics was used for de-multiplexing sequence data into FASTQ files, aligning reads to the human genome (GRCh38), and generating gene-by-cell UMI count matrix. The R package Seurat (v2.0) was used for all subsequent analysis.

Data analysis

Ancestry analysis
We used a reference panel of genotypes and clustering based on principal components to identify the likely ancestry of our 14 multiple myeloma individuals, with an additional 856 Multiple Myeloma Research Foundation (MMRF) cases (including 31 multiple time point cases). We randomly selected 10,000 coding SNPs from minor allele frequency > 0.02 from the 1000 Genomes Project. From that set of loci, we measured the depth and allele counts of each sample's bam using the tool bam-readcount (version 0.8.0). Genotypes were called using these criteria: 0/0 if reference count ≥ 8 and alternate count < 4; 0/1 if reference count ≥ 4 and alternate count ≥ 4; 1/1 if reference count < 4 and alternate count ≥ 8; and ./. (missing) otherwise. After filtering markers with vacancies > 5% in our multiple myeloma samples, 6,349 markers were left for analysis. We performed principal component analysis (PCA) on the 1000 Genomes samples to identify the top 20 principal components. We then projected our multiple myeloma samples onto the 20-dimensional space representing the 1000 Genomes data. To predict the likely ancestry of our multiple myeloma samples, we built a random forest classifier using these 20 principal components, which has known ancestry information for each sample. Using an 80%/20% split between training and test data, our classifier had 99.6% test accuracy. We then predicted the likely ancestry of our multiple myeloma samples based on this classifier.

scRNA-seq data integration
Different scRNA gene expression matrices were integrated using the Seurat R package. We controlled for batch effects using the CCA method and the data were integrated using the top 1000 variable genes from each sample and the first 15 CCs. Cell types were assigned based on manual review of marker gene expression. Cells with inconsistent cell type assignments between the integrated and individual analysis were filtered out. In some cases, the inconsistencies arose from evident clustering issues (for example, when reviewing marker gene expression, two sub-clusters were obvious within one cluster). Such instances were manually resolved and the cells were rescued. All differential gene expression analyses were carried out using the FindMarkers function of the Seurat package. The default Wilcox test was used and hits with adjusted p-value < 0.05 were deemed significant.

scRNA-seq correlation analysis
After integration, for each cell type, we compared the gene expression to other types to identify the significant highly expressed genes (adjusted p-value < 0.05 and log fold change > 0). Then their average expressions in each sample were calculated. Their pairwise correlations were then estimated.

Clustering of sub-populations of plasma cells based on pathway enrichment
We used differentially expressed genes (DEGs, fold change >1.5 and FDR < 0.1) to detect clusters in plasma cells for each sample. We then used the DEGs for each sub-cluster in samples to do pathway enrichment analysis. For the integration pathway analysis, we used the q-value (FDR) associated with each pathway and only used pathways that had at least one significant (FDR < 0.05) association with a cluster in order to filter non-significant pathways. We then calculated the correlation between sub-clusters from different samples based on the 764 pathway FDR values, to see which sub-clusters shared similar enrichment in pathways.

10Xmapping
scRNA data provide an unprecedented resource for studying tumor heterogeneity and clonal evolution. Connecting somatic mutations to individual cells can help to better understand these aspects and have the potential to identify tumor cells which cannot be unveiled purely based on expression data or is difficult to be separated by expression alone. Here, we developed a mapping tool (10Xmapping), which can identify reads supporting the reference allele and variant allele covering the variant site in each individual cell by tracing cell and molecular barcode information in the bam file. The tool is freely available at https://github.com/ding-lab/10Xmapping. For mapping, we used high-confidence somatic mutations from WES data; mutations were combined if data from multiple time points existed.

Single cell RNA CNV detection and clustering
To detect large-scale chromosomal copy number variations using single-cell RNA-seq data, inferCNV (version 0.8.2) 15 was used to obtain relative expression intensity of plasma cells in comparison to a set of reference "normal" cells, including B cells, T cells, Erythrocytes, NK cells, etc. Cutoff=0.1 was used for revealing CNV signals. inferCNV took the raw expression matrix generated from Seurat after several filtering steps, as described above. Subsequently, samples were clustered on inferCNV expression data for 30 genes implicated in MM. Cells for each sample underwent a dimensionality reduction using PCA and t-SNE before clustering. Cells were then clustered with the DBSCAN algorithm. Optimal values for epsilon and minimum points were selected via a grid search. Parameters resulting in the highest Silhouette coefficient were ultimately selected.

Trajectory-based analysis of B cells/Plasma cell lineage
For trajectory analysis, B and plasma cells as a whole were extracted from each case (across time points), respectively. B cell and plasma cells were then imported into Monocle2 74. Parameters for the analysis were consistent with the tutorial (http://cole-trapnell-lab.github.io/monocle-release/docs/#constructing318
single-cell-trajectories), except that (1) cell type is set as the variable for differential expression text and (2) to select genes used for ordering, we set 1e-10 as the q value cut-off. We used the function "plot_cell_trajectory" to visualize B cells and plasma cell subcluster projection in the trajectory. To calculate the proportion of different plasma cell subclusters within each state, B cells and plasma cells that do not belong to any subclusters were removed. The rest of the cells were first normalized by the total number of cells within a time point and then plasma cell subcluster proportions were calculated within each state of interest.

For manuscripts utilizing custom algorithms or software that are central to the research but not yet described in published literature, software must be made available to editors and reviewers. We strongly encourage code deposition in a community repository (e.g. GitHub). See the Nature Research guidelines for submitting code & software for further information.

Data

Policy information about availability of data

All manuscripts must include a data availability statement. This statement should provide the following information, where applicable:

- Accession codes, unique identifiers, or web links for publicly available datasets
- A list of figures that have associated raw data
- A description of any restrictions on data availability

For the corpus of 14 patients, the Washington University Institutional Review Board approved the study protocol, and we have complied with all relevant ethical regulations, including obtaining informed consent from all participants.

All sequencing data (10xWGS, WGS, WES, Bulk RNA-seq and scRNA-seq) used in this study can be accessed at the NCBI under accession code PRJNA694128 [https://submit.ncbi.nlm.nih.gov/subs/sra/SUB8614413/overview]. CyTOF data have been deposited with the FlowRepository (FR-FCM-Z3EP). For ancestry analysis in Supplementary Figure 1b, data was also provided by The Multiple Myeloma Research Foundation (MMRF) CoMMpass (Relating Clinical Outcomes in MM to Personal Assessment of Genetic Profile) Study (NCT01454297). dbGaP Study Accession: phs000748. The MMRF CoMMpass study can be accessed at https://www.ncbi.nlm.nih.gov/projects/gap/cgi-bin/study.cgi?study_id=phs000748.v7.p4.

Field-specific reporting

Please select the one below that is the best fit for your research. If you are not sure, read the appropriate sections before making your selection.

☒ Life sciences ☐ Behavioural & social sciences ☐ Ecological, evolutionary & environmental sciences

For a reference copy of the document with all sections, see nature.com/documents/nr-reporting-summary-flat.pdf

Life sciences study design

All studies must disclose on these points even when the disclosure is negative.

Sample size	The main data corpus of the study comprises 29 longitudinal samples from 14 multiple myeloma patients with different combinations of disease stages, sequencing data types, and treatments (refer to Fig. 1a). All patients have at least one sample with both single-cell RNA sequencing (scRNA-seq) and 10x Genomics linked-read whole genome sequencing (10xWGS), and 9 patients have data from two or more time points, including a mix of CD138+ sorted or unsorted bone marrow aspirate samples. Three patients have data from the SMM and primary stages, and six have both primary and relapse samples. For some samples, whole genome sequencing (WGS), whole exosome sequencing (WES) and bulk-RNA sequencing (bulk-RNA-seq) are also available.<br>Four additional patient samples were included for CyTOF validation experiment.<br>Sample size was determined by the availability of patients enrolled in the study; where bone marrow samples are available, scRNA-seq was determined as the technology to prioritize.
Data exclusions	For scRNA integration, samples with less than 500 cells and samples where only plasma cells are detected are excluded, because they are likely to generate a bias at the all-sample-level integration. Based on this criteria, single cell sample from 37692 SMM, 57075 Primary and 98433 Primary time points are excluded (<500 cells). Sample 25183 Relapse-1 is also excluded (only plasma cells are detected).
Replication	Replication was not included in the study design as human samples are limited.
Randomization	The study design was observational only and did not involve allocating patients into treatment groups. Therefore, randomization was not relevant to the study design.
Blinding	The study design was observational only and did not involve allocating patients into treatment groups. Therefore, blinding was not relevant to the study design.

Reporting for specific materials, systems and methods

We require information from authors about some types of materials, experimental systems and methods used in many studies. Here, indicate whether each material, system or method listed is relevant to your study. If you are not sure if a list item applies to your research, read the appropriate section before selecting a response.

Materials & experimental systems

n/a	Involved in the study
	x Antibodies
x	Eukaryotic cell lines
x	Palaeontology and archaeology
x	Animals and other organisms
	x Human research participants
x	Clinical data
x	Dual use research of concern

Methods

n/a	Involved in the study
x	ChIP-seq
x	Flow cytometry
x	MRI-based neuroimaging

Antibodies

Antibodies used

Antibodies used for CyTOF experiments are listed in Table S6, including manufacture and metals used for conjugation, as well as the target concentration. Antibodies being used and the validation provided by the company are listed below:

CD11c(Bu15,BioLegend; validation from website:Flow Cytometry, CyTOF)
CD123 (IL-3R)(6H6,BioLegend; validation from website:Flow Cytometry, CyTOF)
CD138(DL-101,Fluidigm; validation from website:CyTOF)
CD14(M5E2,BioLegend; validation from website:Flow Cytometry, CyTOF)
CD16(3G8,Fluidigm; validation from website:CyTOF)
CD163(GHI/61,Fluidigm; validation from website:CyTOF)
CD19(HIB19,Fluidigm; validation from website:CyTOF)
CD3(UCHT1,BioLegend; validation from website:Flow Cytometry, CyTOF)
CD34(581,Fluidigm; validation from website:CyTOF)
CD38(HIT2,Fluidigm; validation from website:CyTOF)
CD4(SK3,BioLegend; validation from website:Flow Cytometry, CyTOF)
CD45(HI30,Fluidigm; validation from website:CyTOF)
CD56 (NCAM)(NCAM16.2,Fluidigm; validation from website:CyTOF)
CD61(VI-PL2,BioLegend; validation from website:Flow Cytometry, CyTOF)
CD66b(6/40C,Biolegend; validation from website:Flow Cytometry, IHC-P)
CD69(FN50,Fluidigm; validation from website:CyTOF)
CD71(CY1G4,BioLegend; validation from website:Flow Cytometry)
CD79b(CB31,Fluidigm; validation from website:CyTOF)
CD8a(SK1,BioLegend; validation from website:Flow Cytometry, CyTOF)
CXCR4(12G5,Fluidigm; validation from website:CyTOF)
FCRL5(509f6,Biolegend; validation from website:Flow Cytometry, IP)
H3.3(D1H2,Cell Signaling; validation from website:Flow Cytometry, WB, IHC, IF)
HLA-DQA1(Tu169,Biolegend; validation from website:Flow Cytometry, IHC-F)
IL-10(JES39D7,Fluidigm; validation from website:CyTOF)
IL-10RA(3F9,BD; validation from website:Flow Cytometry)
IL-17(N49653,Fluidigm; validation from website:CyTOF)
IL-17RA(W15177A,BioLegend; validation from website:Flow Cytometry)
IL-1B(AS10,BD; validation from website:Flow Cytometry, IHC)
IL-6(MQ213A5,Fluidigm; validation from website:CyTOF)
IL-6ST(AM64,BD; validation from website:Flow Cytometry)
IL32(373821,R&D; validation from website:Flow Cytometry)
Ig kappa (light chain)(MHK-49,BioLegend; validation from website:Flow Cytometry, CyTOF)
Ig lambda (light chain)(MHL-38,Fluidigm; validation from website:CyTOF)
IgG(97924,R&D; validation from website:Flow Cytometry, CyTOF, WB)
IkBa(L35A5,Fluidigm; validation from website:CyTOF)
Ki-67(B56,Fluidigm; validation from website:CyTOF)
STAT1(SM1,USBio; validation from website:Flow Cytometry, WB, IP)
STAT3(15H2B45,BioLegend; validation from website:ICFC)
TACI(1A1,Biolegend; validation from website:Flow Cytometry)
c-FOS(9F6,Cell Signaling; validation from website:Flow Cytometry, WB, IP, IHC, IF)
c-JUN(60A8,Cell Signaling; validation from website:Flow Cytometry, WB, IP, ChIP, IHC, IF)
p-p38[T180/Y182](D3F9,Fluidigm; validation from website:CyTOF)
p4E-BP1(236B4,Fluidigm; validation from website:CyTOF)
pMAPKAPK2[T334](27B7,Fluidigm; validation from website:CyTOF)
pNF-kB p65[S529](K10-895.12.50,Fluidigm; validation from website:CyTOF)
pStat1[Y701](58D6,Fluidigm; validation from website:CyTOF)
pStat1[Y701](58D6,Fluidigm; validation from website:CyTOF)

Validation

Antibodies are first validated by manufactures where possible. For antibodies where CyTOF validation is not available by the manufacture, or antibodies that are not conjugated with metals for CyTOF experiment, metal conjugation is done first. Then, titration

experiment was conducted to determine the optimum concentration of the antibody, where a maxium signal that does not overspill to the adjacent channels could be reached. The validation of these antibodies is completed during the titration experiments. The validation information of each antibody is provided in Table S6.

Human research participants

Policy information about studies involving human research participants

Population characteristics	Patients were diagnosed with either smoldering multiple myeloma, or multiple myeloma at different stages (primary diagnosis, remission or relapse). All the 14 patients are Caucasians, 10 male and 4 female. The median age at diagnosis was 63 (range 46-69). Eight patients had IgG isotype, 4 being kappa light chain and 4 being lambda light chain, 2 had IgA kappa isotype, 2 had light chain only disease (1 kappa and 1 lambda), and 2 were non-secretory. Five were International Staging System Stage 1, two were Stage 2, 3 were stage 3, and 4 were unreported. The median plasma cell burden by flow cytometry in bone marrow at diagnosis was 24% (range 4-63). By standard fluorescence in situ hybridization (FISH), 1 patient had t (4;14), 3 had t(11;14), and 2 showed del(17p). Four additional patients were included for validation. Two patients have IgG isotype, 1 being kappa light chain and 1 being lambda light chain. One has IgA lambda isotype. One patient has light chain disease (lambda).
Recruitment	Patients were recruited for banking of clinical specimens on a prospective IRB approved protocol. There was no potential self selection bias.
Ethics oversight	Washington University Institutional Review Board

Note that full information on the approval of the study protocol must also be provided in the manuscript.
